# The critical need for a robust research agenda on ultra-processed food consumption and cancer risk

**DOI:** 10.1371/journal.pmed.1004482

**Published:** 2024-11-19

**Authors:** Erikka Loftfield, Steven C. Moore, Susan T. Mayne

**Affiliations:** 1 Metabolic Epidemiology Branch, Division of Cancer Epidemiology and Genetics, National Cancer Institute, National Institutes of Health, Bethesda, Maryland, United States of America; 2 Department of Chronic Disease Epidemiology, Yale School of Public Health, New Haven, Connecticut, United States of America

## Abstract

Ultra-processed food consumption has increased worldwide, but associations with cancer risk remain unclear and potential underlying mechanisms are speculative. A robust, multidisciplinary, research agenda is needed to address current research limitations and gaps.

In recent decades, alongside increasing global availability and consumption of ultra-processed food (UPF) [1], rates of obesity and related chronic diseases have increased. The highest consumption of UPF, defined according to the widely adopted NOVA classification system, is in the United States of America, where, at the population level, UPF accounts for about 60% of calories consumed by adults and children [2,3]. Higher UPF consumption has been consistently associated with obesity in epidemiological studies [4], and obesity is an established risk factor for more than a dozen cancer types [5]. Thus, there is growing concern about the potential impact of UPF consumption on cancer risk. However, critical issues concerning the definition, measurement, and validation of UPF exposures are yet to be resolved. Scientific evidence demonstrating a direct or indirect (through obesity) role of UPF as a hazard in the development of cancer is limited and inconsistent [4], and potential mechanisms linking UPF to cancer development or progression remain speculative.

Consider colorectal cancer (CRC), which, according to the World Health Organization, accounts for about 10% of all cancer cases, making it the third most common cancer and the second leading cause of cancer-related deaths worldwide [6]. In a recent umbrella review, CRC was the only cancer site, of the 6 sites considered, for which an association between higher UPF exposure and higher cancer risk was observed [4]. Of the studies that contributed to this observation, only 3 (out of 7) used a prospective design, which mitigates important sources of potential bias. Each of the 3 prospective studies defined UPF according to NOVA, but each used a different type of dietary assessment tool. The results were inconsistent, providing no clear answers but raising important questions for future research on UPF and cancer more broadly.

First, how should UPF be defined when studying cancer etiology? By design, the NOVA system classifies foods and beverages based on the purpose and extent of processing without consideration for nutritional content. UPF is often described as typically being low in fiber, micronutrients, and phytochemicals [1] despite the fact that some processing technologies can concentrate and enhance bioavailability of compounds such as phytochemicals [7]. In addition, major sources of dietary fiber and calcium, such as whole grain cereals as well as most breads and yogurts, which are associated with lower CRC risk, are categorized as ultra-processed just like most processed meat, which has been associated with higher CRC risk. Thus, this approach ignores scientific consensus, based on decades of research on diet and CRC [8], and could be limiting our ability to identify causal mechanisms underlying potential UPF-cancer associations. At the same time, researchers are understandably averse to changing the NOVA definition of UPF to fit an individual study hypothesis, as lack of clear standardized definitions and inconsistent application of NOVA are criticisms of the field.

Another question that emerges is how to measure and validate UPF consumption. Within and across epidemiological studies, consistency in applying NOVA classifications can vary, and different units of measurement can yield inconsistent results. Most cohort studies, with sufficient follow-up time for studying cancer risk, used food frequency questionnaires (FFQs) to measure long-term, usual dietary intake, whereas the NOVA system was developed using more detailed data from 24-h dietary recalls [2] and is better suited to assessment tools that capture brand name data that are linked to ingredient labels. At a minimum, within cohort studies, validation of FFQs or other questionnaire-based UPF measures is needed to assess questionnaire performance, to inform interpretation of study results, and to compare with other studies using different dietary assessment tools.

Still, advancing research on the industrialized food supply and human health goes beyond consistent application and validation of the NOVA classification system to existing cohort data. The scope of the measurement issue comes into view when we consider that there are thousands of substances allowed in human food; for example, in the USA, this list includes direct food additives, indirect food additives such as food contact substances, and substances that are allowed as “Generally Recognized as Safe” (GRAS) by the Food and Drug Administration (FDA) [9]. Food additives that are of no or rare culinary use or intended to make a product palatable (e.g., emulsifiers, colorants, artificial sweeteners) qualify a product as UPF, but other food additives with preservative functions do not. Furthermore, the combination of ingredients and additives (i.e., formulation) is another aspect of UPF that may independently impact health. Ultimately, studying what is in the food supply and what people are consuming is a big data challenge. Navigating this challenge will require novel, accessible tools, including brand name databases with linkage to nutritional, ingredient, and processing data. Additionally, innovative approaches, including machine learning [10] for NOVA classification and identifying patterns within the heterogenous array of UPF are needed to aid in the discovery of potential mechanisms linking UPF consumption to cancer risk.

Hypothesized mechanisms can be broadly grouped into 2 categories: metabolic and chemical. Suspected metabolic mechanisms underlying potential UPF-cancer associations center around weight gain and obesity. Obesity is a risk factor for multiple cancer types and can be causally linked with several biological mechanisms involved in cancer development including changes in inflammation, sex hormone metabolism, and insulin and insulin-like growth factor signaling [5]. Characteristics of UPF that could contribute to excess energy intake, weight gain, and obesity include higher energy density, lower nutrient density, hyper-palatability, and faster eating rate [11]. Additionally, there is epidemiological evidence to suggest that, at the population level, UPFs are displacing intake of unprocessed and minimally processed foods [2], including whole food sources of nutrients. A multitude of hypotheses could potentially link individual food additives to cancer etiology through a variety of biological mechanisms ranging from alterations in the gut microbiome to endocrine disruption. However, prospective human studies exploring the temporal association between UPF intake, potential metabolic and chemical mediators, and cancer risk are lacking.

Advances in high-throughput technologies, including metabolomics and proteomics, as well as in analytics, including machine learning approaches [10], present opportunities to complement and extend our current understanding of how complex exposures, like UPF, contribute to the etiology of complex diseases, like cancer. For example, we have shown in the context of a randomized, crossover, controlled-feeding trial that consuming a dietary pattern high in UPF, compared to one void of UPF, has a measurable impact on circulating and urine metabolites [12] that could individually or collectively serve as candidate biomarkers of specific foods and beverages or dietary patterns high in UPF. Developing “-omics” signatures predictive of UPF consumption in population-based studies of adults with varying dietary patterns has the potential not only to address some concerns about how to measure UPF consumption but also to provide novel insight into the aspects of UPF (e.g., food additives or food contact substances) and mechanisms underlying potential associations with cancer risk.

Given the complexity of UPF consumption as an exposure and the lack of strong and consistent evidence supporting a causal link with cancer risk, there remains a critical need for a robust research agenda that could help support future regulatory and public health actions. In the meantime, consumers are best advised to follow current dietary guidance for cancer prevention including consuming diets high in whole grains, vegetables, and beans; limiting intake of alcoholic beverages and red and processed meats; and relying on Nutrition Facts labels and Front-of-Pack Nutrition labeling (where available) to avoid processed foods and beverages that are high in added sugars, sodium, and saturated fat [8]. Dietary patterns based on this advice will limit UPF consumption while being supported by decades of underlying scientific research.
